# qPortal: A platform for data-driven biomedical research

**DOI:** 10.1371/journal.pone.0191603

**Published:** 2018-01-19

**Authors:** Christopher Mohr, Andreas Friedrich, David Wojnar, Erhan Kenar, Aydin Can Polatkan, Marius Cosmin Codrea, Stefan Czemmel, Oliver Kohlbacher, Sven Nahnsen

**Affiliations:** 1 Applied Bioinformatics, Center for Bioinformatics Tübingen, University of Tübingen, Sand 14, 72076 Tübingen, Germany; 2 Quantitative Biology Center (QBiC), University of Tübingen, Auf der Morgenstelle 10, 72076 Tübingen, Germany; 3 Max Planck Institute for Developmental Biology, Max–Planck–Ring 5, 72076 Tübingen, Germany; Swiss Institute of Bioinformatics, SWITZERLAND

## Abstract

Modern biomedical research aims at drawing biological conclusions from large, highly complex biological datasets. It has become common practice to make extensive use of high-throughput technologies that produce big amounts of heterogeneous data. In addition to the ever-improving accuracy, methods are getting faster and cheaper, resulting in a steadily increasing need for scalable data management and easily accessible means of analysis.

We present qPortal, a platform providing users with an intuitive way to manage and analyze quantitative biological data. The backend leverages a variety of concepts and technologies, such as relational databases, data stores, data models and means of data transfer, as well as front-end solutions to give users access to data management and easy-to-use analysis options. Users are empowered to conduct their experiments from the experimental design to the visualization of their results through the platform. Here, we illustrate the feature-rich portal by simulating a biomedical study based on publically available data. We demonstrate the software’s strength in supporting the entire project life cycle. The software supports the project design and registration, empowers users to do all-digital project management and finally provides means to perform analysis. We compare our approach to Galaxy, one of the most widely used scientific workflow and analysis platforms in computational biology. Application of both systems to a small case study shows the differences between a data-driven approach (qPortal) and a workflow-driven approach (Galaxy).

qPortal, a one-stop-shop solution for biomedical projects offers up-to-date analysis pipelines, quality control workflows, and visualization tools. Through intensive user interactions, appropriate data models have been developed. These models build the foundation of our biological data management system and provide possibilities to annotate data, query metadata for statistics and future re-analysis on high-performance computing systems via coupling of workflow management systems. Integration of project and data management as well as workflow resources in one place present clear advantages over existing solutions.

## Introduction

As the number of multi-omics projects especially in biomedical research is steadily increasing, the importance of digital management platforms that support the entire project and data life cycle is growing. This is primarily triggered by the explosion in data generated by high-throughput experiments. In particular the daily production of next-generation sequencing data by only one state-of-the-art instrument are in the range of terabytes [1]. This trend is continuing with even more affordable, more accurate, and faster genome sequencing technologies such as Illumina NovaSeq^™^ [2]. Furthermore, multi-omics approaches that aim at assessing biological questions on more than one layer, e.g. simultaneously on genome and proteome level, require sophisticated experimental designs and data integration strategies. As biomedical experiments generally are based on several replicate samples to increase the statistical power, understanding the connection of patients, extracted tissues, and measured raw data is as crucial as recording the experimental variables and biological properties. Additionally, collaborations across different labs or the use of large public datasets such as the *1000 Genome Project* and their meta information often require stringent mapping or conversion of multiple sample identifiers [3]. Tackling all of these challenges is a key requirement for automated data management and analysis in order to pave the way for big data in biomedicine [4]. Furthermore, researchers try to centralize the coordination of these efforts on large-scale research consortia, the International Cancer Genome Consortium (ICGC) being a prominent example. As a result of such biomedical research and infrastructure consortia, impressive collections of biomedical data are made publicly available, building an unprecedented resource for other researchers on a global scale [5, 6]. Therefore, elaborated approaches for standardization of data and experimental metadata storage are needed. Addressing these problems can not only speed up processing of data, but also provide possibilities to check incoming data for correctness and establish automatic processing. The increasing complexity of modern biomedical research makes it difficult for scientists to be involved in every aspect of a project. It is crucial to guarantee a way for different collaborators to access project data coming from different locations. In addition, centralized solutions introduce hurdles on the infrastructure and computational side. Examples are ensuring data access, availability of analysis pipelines for specific omics data, and data security, especially with respect to clinical data. Web-based solutions can bridge the gap between the different parties involved in scientific projects. Such platforms are an established approach to provide scientists with a centralized interface to data, metadata and analysis tools that bring additional value to the project and solve aforementioned problems. In recent years, efforts that try to tackle these problems by developing portal-based solutions have been undertaken for specific biomedical research fields. Clearly, many portal solutions have been developed for domain-specific applications, making a direct comparison difficult. In proteomics, the Swiss Grid Proteomics Portal (iPortal), which itself is based on the Swiss Protein Identification Toolbox swissPIT, provides a web-based solution for identification, quantification and Sequential Windowed Acquisition of All Theoretical Fragment Ion Mass Spectra (SWATH) in proteomics [7–9]. In genomic research there are solutions such as Galaxy, which provide an open web-based platform and workflow system for analysis of genomic data [10]. Another web-based platform which provides analytical tools for gene expression, sequence variation, proteomic, and network analysis among others is GenePattern [11]. Such platforms can also be augmented with additional data and metadata management strategies and converge into a Laboratory Information Management System (LIMS) [12] solution or even larger automated systems [13]. For more specific areas (e.g., cancer research), projects such as the cBio Cancer Genomics Portal are available [14]. cBio Portal, in particular, has a unique focus on visualizing large scale cancer genomics data. Since medical research requires more attention with respect to data privacy and integration of heterogeneous data, specialized solutions such as mediGRID have been implemented [15]. Other areas of research are covered by web-based platforms, for instance, the Molecular Simulation Grid (MoSGrid), which allows users to perform computations in the context of computer-aided drug design (e.g., docking and virtual screening) [16], the neuroscience gateway e-BioInfra [17], or web-based solutions for phylogenetic analyses [18]. The fundamental requirement for annotating new experimental data presented in web portals can also drastically improve the means for sharing data with a wider scientific community. This re-use provides possibilities for data mining and new big data approaches that benefit from more correlative power of leveraged data [19]. With respect to high-throughput biomedical data, web portals require connections to large-scale computing resources, such as high-performance computing (HPC) clusters, grids, or compute clouds. Workflow systems can provide easy implementation and scheduling of established analysis tasks. Here, the gateway interfaces and workflow systems such as the grid and cloud user support environment (gUSE) can also provide new solutions to scientists that are less familiar with command line tools [20]. The aforementioned portal solutions indicate the large variety of served purposes, ranging from bioinformatics workflow management, management of different omics technologies to visualization applications, e.g. for cancer research.

We present qPortal, a web-based platform with an integrated workflow system providing portlets for convenient creation of large-scale experimental designs and data management. On top of this, qPortal provides rapid and easily accessible quality control and data analysis. qPortal leverages a growing stack of software applications that are loosely coupled. The system facilitates the design of projects and experiments, the access, management, and the presentation of the corresponding data and meta information as well as execution of bioinformatics analysis pipelines. qPortal includes features from some of the previously described platforms, with Galaxy sharing most features and serving similar purposes, but both implement different concepts. In this manuscript we emphasize the distinctive aspects of qPortal in comparison to Galaxy and illustrate the underlying concept in a representative use case.

## Implementation

### Database and data model

The backend of qPortal uses the open Biological Information System (openBIS) [21]. OpenBIS offers mechanisms for storage and management of raw data and its annotations as well as functionality for managing access to data. The software package comprises a raw data store, metadata management via a PostgreSQL database as well as an application server for browsing and managing data and metadata [22]. Data models build the basis for handling big data efficiently. The general openBIS data model concept comprises five distinct hierarchically ordered levels. Access rights are managed on the top level (workspaces), which can contain multiple projects, experiments, samples and datasets. Custom openBIS data models can be defined by creating specific types for experiments, samples and datasets and standard plugins for common biomedical experiment types are already available [23]. Entities of those types can contain multiple user-defined properties. The structured storage of metadata is an essential component of the system; metadata is attached to the representations of both the concrete samples as well as their intangible experiments. Metadata in this context comprise information about the used protocol or similar content. For many scientists in the biomedical field, the use of unstructured data formats or spreadsheets containing additional metadata is common. Some of this data is inherently hard to model, but mostly intuitive to understand for humans. For this reason we also provide a way to upload, display and download unstructured project planning and results data to projects.

### User management

In our system, registered users are stored in an in-house Lightweight Directory Access Protocol (LDAP) server connected to the backend (openBIS) and Liferay, which is used as front end [24]. Therefore, we are using the advantages of a single sign-on (SSO) based solution as already implemented for other Grid web applications and portals [25, 26]. The resource containing user information can be easily replaced by any comparable protocol (e.g. Crowd [27]) compatible with openBIS and Liferay. Data privacy is of high importance, especially in biomedical applications. Access to data and metadata is regulated on different levels. In principle, this process can be further extended to make use of concepts like a two-factor authentication. The primary login mechanism is needed on the Liferay landing page, followed by a delegation mechanism to the back-end database. All available information is added automatically. Each user or defined user group can be assigned multiple roles, regulating access to openBIS spaces that might include several projects and the corresponding data. Therefore users are only able to access data connected to projects in spaces to which they have been granted access.

### Data transfer

Data transfer from different locations to the central data repository is realized using rsync, as implemented in the openBIS Datamover [24, 28]. Data is synced from the source, e.g., the genome sequencing machine to defined folders on a remote storage. Checksums allow monitoring of data integrity while transferring data to the final catalogue via openBIS dropboxes according to their source and data type. Every dropbox implements an Extract Transform Load (ETL) routine. These scripts are based on Jython and handle the raw data and possibly connected metadata files. Depending on the needed ETL process, additional external scripts can be called. Since more information is collected when actually preparing the samples and measuring the data, the portlet responsible for experimental design (Project Wizard) only registers the main information of the experiment and meta information of the project. ETL scripts complete this task by creating new entities like experiments, samples and datasets and finalize the experimental model by extracting metadata from incoming files. This information is then stored as content of defined properties of the new entities that are then also connected to existing entities in the database through defined identifiers. The raw data and possibly converted files are then connected to datasets and moved to the data store.

### Workflow system

gUSE enables the portal users to perform computations on cluster infrastructures, automating common bioinformatics workflows and data analysis steps. The technology enables access to distributed computing infrastructures (DCIs) and provides a graphical user interface through WS-PGRADE, which can be used to create new workflows and manage existing workflows. Therefore workflows that are not yet available can be created by users using their WS-PGRADE instance before being ported to a qPortal instance. Due to the modularity of available workflows (see Suppl. S1 File for the comprehensive list of workflows) and fine tuning via changing parameters most of the common analysis tasks can already be performed by subsequent workflow runs. Additionally, by creating an interface in the Workflow Handler java library, the gUSE workflow system can be replaced by other workflow systems like Nextflow [29]. The status of a workflow is defined by the gUSE workflow status and the according openBIS experiment status, which is updated when a workflow has been submitted or results have been registered. This interface to the gUSE functionality has been implemented in qPortal as VAADIN-based portlets [30]. After submission, gUSE workflows are scheduled on the cluster by a submitter. The running instance is using a 2x10 Gigabit Ethernet connection and 2x Infiniband Mellanox providing speed up to 56 Gbit/s. The servers of our current infrastructure are split up as follows: 1 NFS server with 64 GB RAM, 3 virtualization nodes (250 GB RAM), two high memory nodes for computation with 512 GB RAM each and 25 computational nodes with 64 GB RAM each. Since the server system schedules jobs submitted through qPortal, there is no limitation regarding the number of jobs submitted on the portal, but for jobs running in parallel. However, with gUSE as our production workflow management system, the applicability of the portal is not limited to an infrastructure as exemplified above, but can be adapted to a diverse collection of compute resources. The current hardware setup connected to our qPortal instance in Tübingen has been extensively used for various biomedical applications like NGS analysis. These compute resources have shown to be sufficient in around 500 conducted projects.

**Fig 1:**
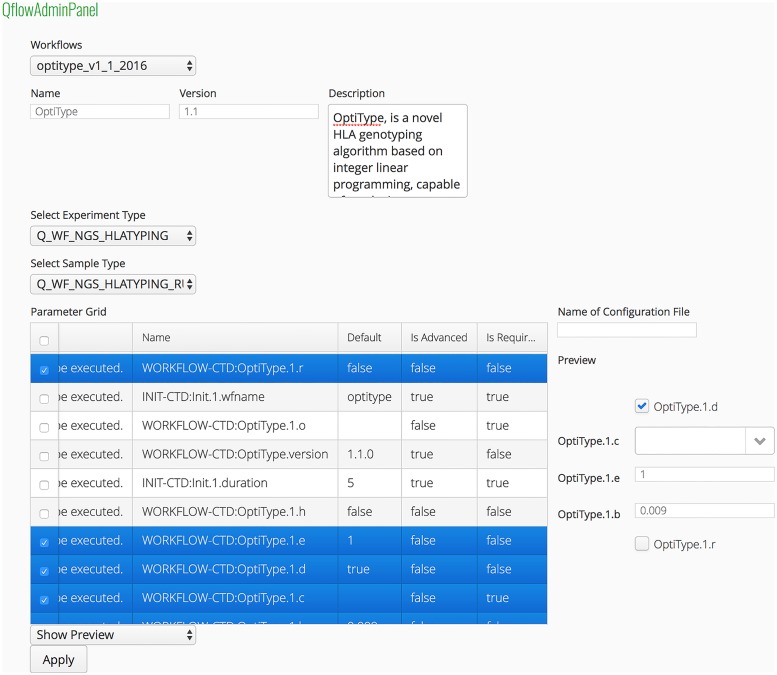
qPortal workflow admin panel. Configuration of workflows is done once through the workflow admin panel in qPortal. Besides the name, version, and a description of the worklfow, associated experiment and samples types in openBIS have to be selected. Workflow runs and their results will be stored in the database as data and meta information. Additionally, the parameters of the workflow which will be shown to the user can be selected (highlighted in blue in this example).

Parameters of workflows are defined by using Common Tool Descriptors (CTDs). CTDs are XML files that can be used to store information about execution of software tools. This information can contain specifications of parameters, input files and output files. CTDs have been previously used in the context of workflow conversion [31, 32]. The configuration of workflows is done once through a configuration portlet in qPortal (Fig 1). Implemented workflows (gUSE) can be imported and configured in the admin panel. The corresponding interface can be extended to other workflow engines. Therefore, qPortal can be easily extended to preferred workflow engines. This portlet offers functionality for setting workflow metadata like the name, version and description as well as choosing workflow parameters that should be shown to the end user in the workflow submission view of the Project Browser. This information is stored in a JSON file, which is used to represent the workflow in the portlet by visualizing the selected parameters and selection of input files accordingly. Based on this information only workflows suitable for the types of data at hand are presented to the user.

### Main features of qPortal

**Fig 2:**
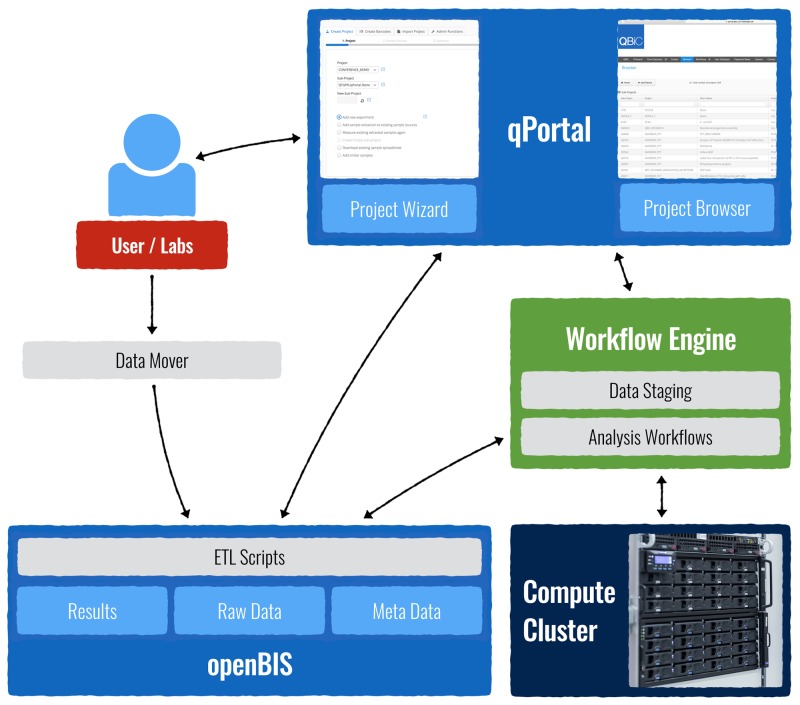
qPortal setup. qPortal is connected to openBIS and the workflow engine. The workflow engine is connected to a high-performance computing cluster. Users may create projects, upload their data (Datamover), and analzye it through qPortal (Project Wizard and Project Browser). Results are automatically written back to the database and presented on the portal (Project Browser).

The qPortal software is built on top of a Liferay 6.2 Instance. It includes a collection of portlets, which are web applications written in Java using the open-source framework VAADIN [30], based on Ajax and Google Web Toolkit. The main portlets, the Project Browser and the Project Wizard are written in Java 1.7 using VAADIN 7. Those portlets are accessible after logging in with the corresponding user credentials in qPortal. The Liferay instance is running on Tomcat 7 [33]. Fig 2 shows the overall setup of qPortal.

#### Project planning

Metadata are an essential component of any data-driven experiment and project. Yet, metadata annotations are still poorly handled in many projects. Sparse metadata deposition and tracking is increasingly becoming a concern that grows with falling costs for data generation [34]. Typically, there are multiple steps involved in the sample preparation and measurement of a biological experiment. Each of these steps in the whole process is associated with a collection of structured and unstructured metadata. To enable fast and intuitive project planning we developed the Project Wizard portlet that leads the user through a series of well-defined experimental steps [35]. In order to keep track of samples and data related to these steps, we use a customized data model as previously described based on a multi-tier experiment: the first step describes biological entities such as patients, model organisms or any other biological system of interest. Users are supported by a controlled vocabulary taken from the NCBI Taxonomy database [36]. The second step describes the extraction of cells or tissues from the aforementioned biological systems. The last step describes the preparation of the samples analyzed by the actual data collection systems like RNA or DNA for next generation sequencing, protein preparations for mass spectrometry or other methods. At each of these steps, other meta information, such as drug treatments, genotypes or cell culture growth conditions can be added to the defined samples. Additionally, every sample type also allows the addition of distinctive information, e.g. internal lab identifiers to connect samples to other databases, a human readable description of the sample or other additional information concerning the sample. As a part of qPortal, the Project Wizard has been implemented using the Java VAADIN web library and is based on a full-factorial design: in each of the aforementioned steps, cases of study variables and replicates are multiplied to create sample representations in the system and simplifying the input of replicate data. Since not every experiment consists of the same number of replicates for each case, the user can remove superfluous samples. For complex experiments, input via uploaded experimental design spreadsheet is also possible. All these metadata and relations between samples are then stored in an openBIS instance. The hierarchical model of openBIS allows the representation of biological experiments and samples in an intuitive way. Although properties for different sample and experiment types have to be defined in advance, all types are connected to an XML-based property in our data model implementation, which offers the possibility to extend the amount of metadata that should be stored with entities in a generic manner. These XML properties can be controlled by providing XML schemata that are used to present users with a robust option to record a diverse set of meta information as found in modern biomedical experiments. Other advantages of these schemata are easy parsing and on-the-fly validation of the input, since the strucutred information must conform to the correct document type definition of the schema. This facilitates the input of correct meta information and helps prevent the common problem of missing meta data. To match the sample and experiment representations in openBIS to the newly generated data, a unique identifier is created for each sample. Barcodes encoding those identifiers allow for easy tracking of samples and corresponding machine-created data. The barcodes use a weighted checksum digit for error detection and can be provided for sample tubes as well as on printable sample sheets. They are ultimately scanned to name the data files automatically before transferring them to the data center.

#### Project management and workflows

**Fig 3:**
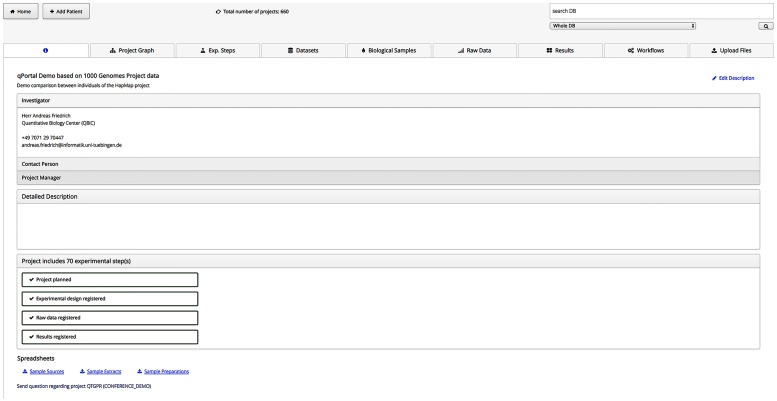
Project view in qPortal. The main view of the Project Browser shows general information about the project such as the name, description, investigators, and a status. Users can navigate to other views like the project graph, available datasets, and the workflow submission view by clicking on the corresponding tabs.

The portlet for project browsing in qPortal is implemented using VAADIN. In the home screen all projects on behalf of the currently logged-in user are shown in a table, providing the project code, the workspace and some description, which can be filled in when the project is registered and edited later. By clicking on the corresponding project, users are forwarded to the project view showing all information about the specific project in a horizontal view with different tabs (Fig 3). The first tab shows general information about this project as the principal investigator and the status of the defined experimental steps. Information about principal investigators and persons responsible for experiments are stored in a MySQL database. The second tab shows the project graph, which is generated using a Java graph library representing the first four layers of the project as registered in the database. Therefore the relationship between samples in different layers and the number of samples can be easily seen. User IDs are queried from openBIS; full names are given by the Liferay instance. All experimental steps and datasets are shown in the next tabs. Datasets can be downloaded from all tables, either as single files or as tar file if multiple datasets have been selected. Tab-separated files, html files and NGS or mass spectrometry quality reports can be directly visualized in the portlet by clicking on the corresponding table row.

In the biological sources and measured samples tab, registered samples of the corresponding layer are shown. Derived samples and their biological sources, such as organisms, soil or water, are shown as well. For measured samples the corresponding raw data, including the file names are shown. The results tab shows datasets that contain results derived from analysis runs in this project. Most of those results originate from workflow runs directly triggered from the portlet, thereby providing a direct trace to the parameter settings used for the generation of these results. Workflows can be submitted in the workflows tab. All workflows for which the corresponding project fulfils the input data requirements are shown with their description and annotated version. After selecting a workflow, input files have to be selected and the parameters, which are shown to users can be set. For parameters that are neither visible to the end user nor changed, default values are used. Every gUSE workflow contains two data staging nodes. In the first initialization node, selected data is copied over to the newly created workspace. The last node commits the result files to the corresponding openBIS dropbox, which is defined in a JSON-based configuration file. In case of existing workflow implementations of another type, they can be easily integrated into the main workflow node. For example, some of the available analysis pipelines include Snakemake workflows [37]. In the upload files tab it is possible to upload small files, unstructured data that is directly attached to this project. Unstructured data might be results or files regarding the planning stages of a project.

**Fig 4:**
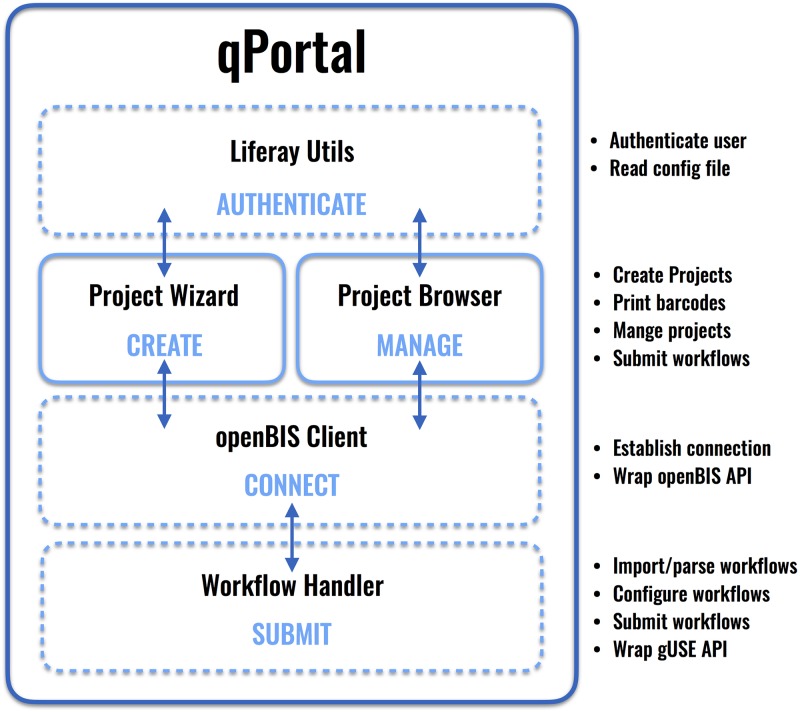
qPortal software components. qPortal consists of several software components which have been implemented. The Java library Liferay Utils is connected to each portlet and ensures authentication of users and enables Liferay functionality for our portlets Project Wizard and Project Browser. These provide users with means of project creation and management via another Java library wrapping the openBIS API (openBIS Client). Import, configuration and submission of workflows via the communication with the gUSE API and openBIS is handled by our implemented Java library Workflow Handler. Java libraries are shown in dashed line boxes whereas the two presented main portlets of qPortal are shown in regular boxes.

The implemented components of qPortal, their interconnectivity and features of the main components, Projct Wizard and Project Browser, are shown in Fig 4.

## Results

### Case study

**Fig 5:**
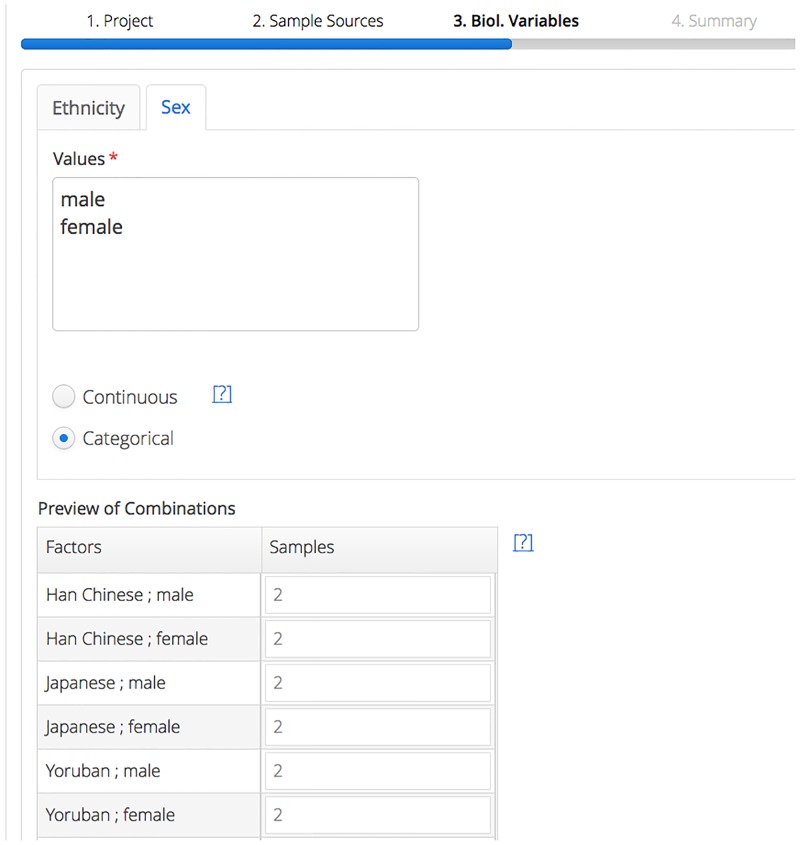
Project registration. One part of the project registration through the Project Wizard as first step of the project management workflow realized by qPortal: values of the experimental design variables ethnicity and sex are input by the user. Resulting combinations are shown and can be adjusted by the user.

To demonstrate the features of qPortal, we registered samples of 20 individuals of the 1000 Genomes Project [38]. We selected the datasets of male and female individuals of 10 different populations, using the annotation in the 1000 Genomes Project (see Data in Table S2 File for sample IDs). A new project was registered with the Project Wizard portlet to add information about the individuals, as well as the extracted blood and DNA samples. Both of the registered characteristics (i.e. sex and population) were selected as experimental variables on sample source level. Details of the registration process can be seen in Fig 5.

**Fig 6:**
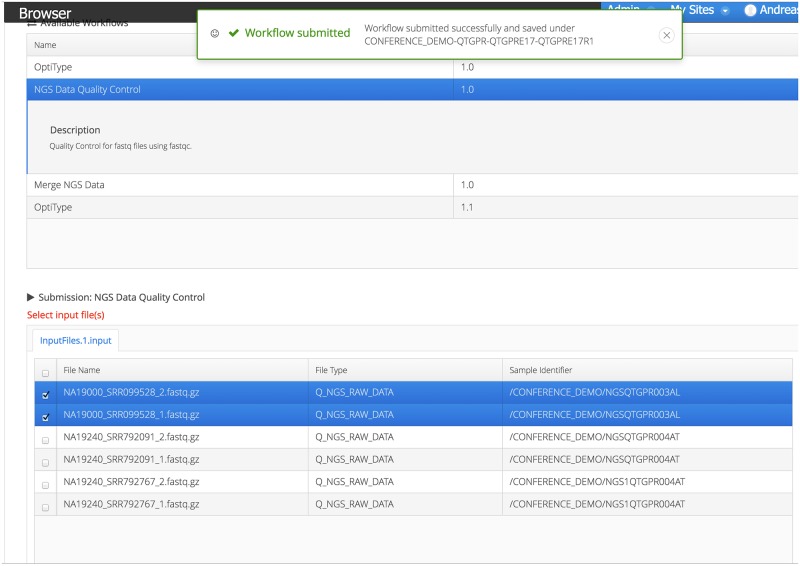
Workflow selection and submission. All available workflows for the corresponding project can be seen in the workflow tab. After selecting the workflow, users select input files and specify the parameter values. If the submission was successful, users will be notified.

Furthermore, we selected one NGS exome-sequencing run for 19 of the individuals and two runs for one individual. The automatically created identifiers were mapped to the IDs of the 1000 Genomes Project. In the lab, this step happens at the time of data creation using provided barcodes. The dropbox system for genomic raw data registration was then used to move the data to our storage and the ETL process automatically performed the association of raw and metadata. Technical replicates of aforementioned runs were detected by the ETL script resulting in 21 NGS samples with meta information being added to the created project. Subsequently, we performed quality control using the FastQC workflow on the sequenced reads of all samples using the workflow tab in Project Browser [39]. After selection of the workflow, input files are selected and the the values of the parameters are specified (Fig 6).

**Fig 7:**
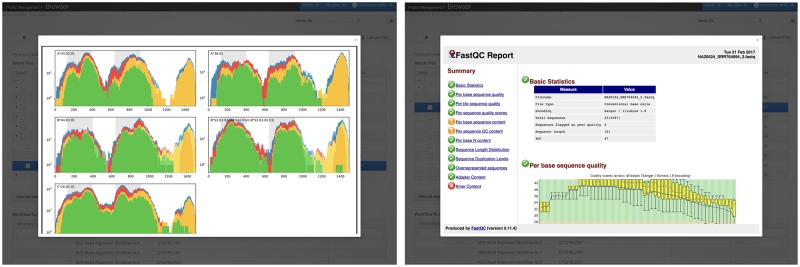
Workflow result presentation. Results of workflow runs will be directly visualized in qPortal. In this case results of one OptiType and one FastQC run are visualized.

The resulting quality control reports in HTML format can be downloaded or directly visualized in the Project Browser. To demonstrate more elaborate NGS data analysis on the portal, we performed NGS read mapping using BWA [40] against the human reference genome ([41], GRCh37; hg19 Feb. 2009 assembly) for two individuals. Furthermore, we used the aligned reads to determine the human leukocyte antigen (HLA) type for the two patients applying a workflow using OptiType, a tool for precision HLA typing from NGS data [42]. For the remaining 19 individuals we performed HLA typing directly on the registered raw data in FASTQ format. The HLA type is crucial for state-of-the-art biomedical applications, especially in the field of personalized medicine. All workflow results are directly visualized in the portal as seen in Fig 7 and are further added as metadata to the project.

### Advantages of a data-driven research platform

To assess usability of qPortal, we conducted a comparison with galaxy instances and provide below the added value of the data-driven qPortal approach. The focus here is on specifying the experimental steps and annotation early and leverage this information throughout the whole project life cycle to facilitate analysis.

One main aspect of qPortal is **project management**. This includes the functionality to register and store projects with customized **experimental designs** and functionality to maintain them. This property is essential to define the data-driven approach. The annotation of data is the primary focus throughout the entire life cycle of experiments and projects. These features are provided by qPortal’s web applications Project Wizard and Project Browser. Experiment and sample instances representing the experimental design are registered and used to attach incoming data. In contrast, the clearly workflow-driven approach of Galaxy is not built upon this concept, but uses implicit project management with a focus on file-based analysis and visualization.

**Data annotation** is possible in both systems, however the emphasis is different. As previously introduced, metadata can be entered in qPortal along with general project information and experimental design upon registration or added afterwards through Project Browser. Notes explaining certain steps of an experiment or special characteristics of samples can be added. Galaxy offers two very similar concepts in that users can add notes and tag items to make them searchable [10]. While qPortal collects metadata for every step of the experiment, there is a clear focus on the annotation of workflow runs in Galaxy. The latter property is essential for reproducibility as well as benchmarking of different workflows and parameters. The qPortal approach of starting extensive metadata collection before the experiment is performed has numerous advantages. Firstly, time and money can be saved, because the study design allows for estimation of statistical power before experiments are performed. Secondly, mistakes in study design or sample handling can be traced back more easily and with higher confidence.

**Data import** is another important criterium regarding usability, especially in biomedical applications. Data import to qPortal is possible using the Datamover feature. Automatic registration including ID mapping and file format recognition is done by ETL scripts and is built upon the experimental design and connected barcode creation. This can be file-type or lab-specific, in the last case often containing additional metadata annotation to be registered. For small, unstructured data a project-specific upload through the browser is available. Galaxy offers functionality for direct data upload through the web browser with varying rules and governance as defined by the Galaxy instance provider. Upload of larger files is supported via encrypted FTP. Additionally, data transfer of input data directly from provided URLs is possible. The Galaxy upload provides auto detection of many commonly used file types. More extensive operations on the input data, comparable to our ETL scripts, could be implemented by the user in the form of workflow nodes.

Due to the data-driven concept of qPortal, users will only be able to see **workflows** for which required input data is available. This data is selected from the corresponding project and parameters of workflows can be easily adjusted. In general, Galaxy offers functionality to adjust parameters. However, as in qPortal, details depend on the implementation of workflows. In the current version, qPortal does not offer users functionality to create workflows in the portal. Instead, custom workflows created in WS-PGRADE can be ported. Another crucial difference is that the qPortal workflow system can make use of the registered experimental design information and other annotations, for example to color graphical analysis results according to different study variables.

**Results** of workflows are automatically registered in the corresponding project in qPortal. Users can download or directly visualize results in the web-based portal. Visualization and download functionality is available as well in Galaxy.

Since biomedical data is normally bound to strictly regulated terms regarding **data security**, access and confidentiality, qPortal utilizes the rule based permission scheme of openBIS. Users have to login and will only be able to access their own projects or the ones of collaboration partners, including the corresponding data. Data transfer is done by Datamover using ssh connections. In Galaxy, registered users are able to upload data to their own workspace or share it with other users.

## Discussion

qPortal, a web-based platform, facilitates data management and empowers scientists to integratively analyze and navigate through large-scale biological data. We presented qPortal’s Project Wizard to perform factor-based experimental designs and register complete biomedical research projects to a tailored database. ETL processes build the backbone of the integrated data management system. Full automation can be achieved in adding data and metadata to registered experiments. Using ETL processes to call complex external scripts, automation can be further supported, for example to convert raw vendor formats for mass spectrometry data into the open format mzML for further analysis [43]. The Project Browser portlet allows project navigation and management for end users. The functionality includes project status monitoring and access to all data of the project. Commonly, modern research projects are coordinated within larger consortia, implying distributed data generation and many stakeholders. The web-based nature of our approach facilitates the implementation of qPortal as a central platform in such projects. Easy data sharing and remote communication in the context of scientific projects are receiving more and more attention and portals, such as qPortal can provide such solutions. Additionally, the workflow-based analysis module provides end users with intuitive interfaces to powerful compute resources allowing for easy execution of bioinformatics pipelines. This way, the complexity of distributed computing infrastructures is hidden from the end user and thereby enables data analysis for scientists without prior scripting or command line experience. With the continuously growing number and throughput of omics technologies, the need for full automation in data management and analysis is obvious. The generic design and the flexible open-source components of qPortal allow for easy adaption and extension. Extending the data model and automatic data registration for additional data types are continuous requests. To guarantee flexibility, we developed our software using the web-framework VAADIN, which has an active developer community providing useful tools and add-ons. It is based on the Google Web Toolkit, providing a wide range of browser support. We further chose Liferay as a free and open source enterprise portal solution. Liferay has an active community and offers packages to add additional features to a portal installation. Since Liferay implements the Java Portlet Specification (JSR 168), which standarizes how portlets interact with portlet containers, compatibility across different portal products is ensured [44]. This means that the qPortal components can run on other portal systems like the JBoss Portal, as long as they are JSR 168 compliant, with minimal effort [45].

There have been comparable efforts towards web-based platforms in biomedical research, like Galaxy. Galaxy facilitates bioinformatics analyses for scientists without expertise in programming and scripting. Galaxy users do not need to install programs or use the command line. Additionally, Galaxy instances frequently provide free storage and compute resources and it is easy to share analysis results with collaborators. With respect to means of workflow submission and the prevention of issues due to infrastructure, programming and scripting, qPortal provides similar features as Galaxy, although the integration level for many tools and different infrastructures in Galaxy is well established. qPortal’s interface to workflow systems is generic by design. While the current production instances interface with gUSE/WS-PGRADE, the extension of the workflow system with interfaces to SnakeMake and NextFlow is possible. Therefore, users will be able to create SnakeMake or NextFlow workflows and use them through qPortal provided that the corresponding tools are available on the cluster instance. Newly developed interfaces can make us of the existing implementations for data staging. Given a network connection between the portal, the workflow system and openBIS, the existing tools for data staging have to be incorporated to the workflows itself. New workflows might entail the extension of the underlying openBIS data model if corresponding experiment, sample and dataset types are not yet existing.

In addition, qPortal provides features for experimental design, metadata handling, project management and collaboration. The focus of Galaxy on workflow annotation aims primarily at reproducibility of computational analyses. In fact, numerous studies in recent years strongly support the importance of this approach. Annotation of analysis pipelines and keeping track of the used parameter settings is crucial in order to help solve the reproducibility issue [46–48]. The comprehensive data-driven approach of qPortal takes the notion of reproducibility one step further. Starting with extensive metadata collection before the experiment has numerous advantages for scientific studies. Mistakes in study design or sample handling can be traced back more easily and with higher confidence. Time and money can also be saved, because the study design allows for estimation of statistical power before experiments are performed. In addition, well annotated experimental data is much more likely to be reused in future research. Other studies support this approach and note that without sound experimental design, even computationally reproducible results have to be used with caution [49]. To increase confidence in a scientific hypothesis, studies must also be replicable when using the same experimental setup to generate new data. Causes for a lack of this replicability mainly include missing statistical considerations, poor experimental design or missing information about the used protocols [50, 51].

While qPortal (as Galaxy) implements thorough logging of processing, parameters, and pipelines, qPortal further facilitates the data annotation, which is equally important for reproducible research and thereby follows the FAIR guiding principles for scientific data management [52].

Galaxy provides intuitive means for data upload, but use-cases often require secure means that are not channeled via the public Internet. Potential security leaks can be easily circumvented by using qPortal’s data moving functionality.

## Conclusion

Here, we present qPortal, a scalable system for the entire life cycle in modern data-driven biomedical research. The XML-based metadata modelling provides the potential for full automation in data processing, since dedicated workflows make use of the structured metadata from the system. We anticipate that web platforms, such as qPortal, will build cornerstones for state of the art research infrastructures. The scalable nature of the setup allows the integration of large in-house and public datasets and thereby builds an ideal ecosystem for big data analysis in biomedicine.

### Availability and requirements

qPortal is licensed under GNU General Public License as published by the Free Software Foundation, either version 3 of the License, or any later version, as published by the Free Software Foundation [53]. Our software and a documentation can be found on http://qbic.life/software. The documentation includes a manual on how to set up qPortal at your site and direct links to the corresponding GitHub repositories.

Our qPortal instance can be accessed on http://qbic.life. The datasets analysed during the current study are available in the 1000 Genomes Project data portal [54]. All data analysed during this study are included in a previous article [38].

## Supporting information

S1 File List of workflows. List and description of workflows available through qPortal. For every workflow the used software is listed.
(DOCX)

S2 File 1000 Genomes IDs. Table (.xlsx) containing the sample identifiers of the 20 samples used, the experimental variables ethnicity, sex of the respective subjects, and information about technical replicates.
(XLSX)

## Abbreviations

CTD: Common Tool Descriptor
DCI: Distributed Computing Infrastructure
ETL: Extract Transform Load
gUSE: Grid and Cloud User Support Environment
HLA: Human Leukocyte Antigen
HPC: High-Performance Computing
ICGC: International Cancer Genome Consortium
LDAP: Lightweight Directory Access Protocol
LIMS: Laboratory Information Management System
MoSGrid: Molecular Simulation Grid
openBIS: open Biological Information System
SSO: Single Sign-On
SWATH-MS: Sequential Windowed Acquisition of All Theoretical Fragment Ion Mass Spectra
XML: Extensible Markup Language
