# Supplementary material for: qPortal: A platform for data-driven biomedical research
Source: PLoS One. 2018 Jan 19;13(1):e0191603. doi: 10.1371/journal.pone.0191603 (PMC5774839; doi:10.1371/journal.pone.0191603)
Supplement: S1 File — List and description of workflows available through qPortal. For every workflow the used software is listed. (DOCX) [file pone.0191603.s001.docx]

Workflows available through qPortal

1. EPAA - Epitope Prediction and Annotation (v1.0)
2. Epitope Prediction (v1.0)
3. Individualized Proteins (v1.0)
4. Individualized Proteins (v2.0)
5. Ligandomics QC (v1.0)
6. Ligandomics ID (v1.0)
7. OptiType (v1.0)
8. OptiType (v.1.1)
9. Somatic Variant Calling (v1.0)
10. Variant Calling (v1.0)
11. Variant Annotation (v1.0)
12. Variant Annotation (v2.0)
13. 16S Taxonomic Profiling (v1.0)
14. Merge NGS Data (v1.0)
15. NGS Quality Control (v1.0)
16. NGS Read Alignment (v1.0)
17. RNA-seq (v1.0)
18. Differential Expression Analysis (v.1.0)
19. shRNA Counting (v1.0)
20. MaxQuant (v1.0)
21. Quality Control (LC-MS based Proteomics; v2.0)
22. OpenMS Peak Picking (v1.0)
23. X!Tandem Peptide ID (LC-MS based Proteomics; v0.3)
24. Microarray Quality Control (v0.8)

## EPAA - Epitope Prediction and Annotation (v1.0)

Pipeline for prediction of MHC class I and II epitopes from variants or peptides for a list of specified alleles. Additionally predicted epitopes can be annotated with protein quantification values for the corresponding proteins or differential expression values for the corresponding transcripts.

Workflow-Software (in order):

- FRED2 (python package)
- netMHC 4.0
- netMHCpan 3.0
- netMHCII 2.2
- netMHCIIpan 3.1

## Epitope Prediction (v1.0)

Epitope Prediction for a list of annotated variants and specified MHC class I and II alleles.

Workflow-Software (in order):

- FRED (python package)
- netMHC 3.0
- netMHCpan 2.4
- netMHCII 2.2
- netMHCIIpan 2.0

## Individualized Proteins (v1.0)

Workflow for generation of protein sequences based on mutations. Mutations will be introduced in the corresponding transcripts and translated to proteins.

Workflow-Software (in order):

- FRED

## Individualized Proteins (v2.0)

Workflow for generation of protein sequences based on mutations. Mutations will be introduced in the corresponding transcripts and translated to proteins

Workflow-Software (in order):

- FRED2

## Ligandomics QC (v1.0)

Workflow for performing quality control on (HLA) ligandomics data. The result will be a qcML file.

Workflow-Software (in order):

- openms 2.0-44ed56b
- netMHCpan 3.0
- netMHCIIpan 3.1
- comet 2015024
- R 3.2.2

## Ligandomics ID (v1.0)

Workflow for performing identification on HLA ligandomics data. The result will be an idXML file and a tab separated file. Additionally, the output will be filtered according to the given FDR value. The complete list will be available as well.

Workflow-Software (in order):

- openms 2.0-44ed56b
- netMHC 3.4
- comet 2015024
- R 3.2.2

## OptiType (v1.0)

OptiType, is a novel HLA genotyping algorithm based on integer linear programming, capable of producing accurate 4-digit HLA genotyping predictions from NGS data by simultaneously selecting all minor and major HLA-I alleles.

Workflow-Software (in order):

- OptiType
- razerS
- cbc

## OptiType (v1.1)

OptiType, is a novel HLA genotyping algorithm based on integer linear programming, capable of producing accurate 4-digit HLA genotyping predictions from NGS data by simultaneously selecting all minor and major HLA-I alleles. This workflow is using the most recent version of OptiType and is capable of dealing with BAM files.

Workflow-Software (in order):

- OptiType
- Yara

## Somatic Variant Calling (v1.0)

Somatic variant calling for tumor and normal tissue samples using Strelka. Strelka is an analysis package designed to detect somatic SNVs and small indels from the aligned sequencing reads of matched tumor-normal samples [Saunders et al., 2012].

Workflow-Software (in order):

- Strelka 1.0.14
- VCFlib 0.1
- samtools 1.3

## Variant Calling (v1.0)

Workflow for variant detection using FreeBayes, a bayesian genetic variant detector. Used for variant calling if there is no tumor-normal comparison to do or if there is no normal sample for tumor analysis available.At the end of the workflow variants are annotated with snpeff.

Workflow-Software (in order):

- NGS-bit - SeqPurge (quality control of raw fastq data: adapter removal, clipping based on quality)
- Bwa-mem (mapping)
- GATK / Picard tools to realign indels, mark PCR/optical duplicates
- FreeBayes 0.9
- Samtools 1.3
- NGS-bits
- VCFlib
- BCFtools
- VCFtools

## Variant Annotation (v1.0)

Annotate variants in vcf format using ANNOVAR. Output includes a file in a format which can be used as input for the Epitope Prediction Workflow.

Workflow-Software (in order):

- ANNOVAR

## Variant Annotation (v2.0)

Annotation of genetic variants in Variant Call Format (VCF) using SnpEff [Cingolani, 2012]. The workflow will generate an annotated vcf file and a file in a format which can be used as input for the Epitope Prediction Workflow.

Workflow-Software (in order):

- snpEff

## 16S Taxonomic Profiling (v1.0)

Taxonomic profiling for 16S metagenomic samples using MALT.

Workflow-Software (in order):

- Clipandmerge 1.7.5
- MALT 0.3.8

## Merge NGS Data (v1.0)

This workflow can be used to merge reads in fastq format for one sample coming from different lanes.

Workflow-Software (in order):

- Bash

## NGS Quality Control (v1.0)

Quality Control for NGS reads.

Workflow-Software (in order):

- FastQC 0.11.4

## NGS Read Alignment (v1.0)

Alignment of NGS reads against a specified reference genome.

Workflow-Software (in order):

- samtools 1.2
- bwa
- FastQC 0.11.4
- Picard
- NGS-bits

## RNA-seq (v1.0)

RNA-seq pipeline using TopHat2, a gapped-read mapper for RNA-seq data and HTSeq for providing feature counts.This pipeline produces raw read counts which are the desired input for differential expression tools for count data such as DESeq2 and/or edgeR. This pipeline is designed for single end (SE) and paired-end (PE) sequencing studies.

Workflow-Software (in order):

- FastQC 0.11.4 (Quality control of raw fastq data)
- Cutadapt (adapter removal, quality filtering)
- Tophat2
- Bowtie2 2.2.3
- htseq-count 0.6.1p2 (producing raw counts for features such as genes,exons)

## Differential Expression Analysis (v1.0)

Differential Expression Analysis using DESeq2 for a comparison of two groups (tumor vs. normal e.g.). Analysis is based on count data coming from a RNAseq workflow run. Make sure to select the count text files of the corresponding RNAseq runs.

Workflow-Software (in order):

- R 3.2.2 (including DEseq package)

## shRNA Counting (v1.0)

Workflow for counting shRNA expression. Given a set of reads from shRNA sequencing, we count how often each reference shRNA sequence occurs at the expected position in the reads. Each read should contain a barcode at a specified position. We divide the reads according to those barcodes.

Workflow-Software (in order):

- In house development script (by Adrian Seyboldt)

## MaxQuant (v1.0)

MaxQuant is a quantitative proteomics software package designed for analyzing large-scale mass-spectrometric data sets. It supports all main labeling techniques like SILAC, Di-methyl, TMT and iTRAQ as well as label-free quantification. Also measured spectra of various vendors - Thermo Fisher Scientific, Bruker Daltonics, AB Sciex and Agilent Technologies - can be processed using MaxQuant.

Workflow-Software (in order):

- mqrun
- MaxQuant

## OpenMS Peak Picking (v1.0)

Workflow for performing peak picking on .mzML data. Outputs centroided .mzML data

Workflow-Software (in order):

- openms 2.0-44ed56b

## Quality Control (LC-MS based Proteomics; v2.0):

This workflow is based on Walzer et al., qcML: an exchange format... The Workflow generates a qcML file based on one or several LC-MS runs. The qcML file can be used to assess the quality of the underlying (input) data.

Workflow-Software (in order):

- openms 1.11.1-2996-g13ffbd7
- Xtandem 201309011-gnu-4.8
- R 3.1.0

## X!Tandem Peptide ID (LC-MS based Proteomics; v0.3):

This workflow uses the peptide search engine X!Tandem (http://www.thegpm.org/TANDEM/index.html) to perform the assignment of peptide sequences to MS2 spectra. The whole workflow includes several other openMS (www.openms.de) tools that allow statistical assessment and a false discovery rate (FDR) - based filtering.

Workflow-Software (in order):

- openMS

## Microarray Quality Control (v.0.8):

Workflow to perform some quality control on raw data of a microarray experiment

Workflow-Software (in order):

- R 3.2.2 including packages:
  - Biobase
  - oligo
  - annotation
  - ggplot2
  - dendextend
  - affycoretools
  - genefilter
